# The role of lattice vibration in the terahertz region for proton conduction in 2D metal–organic frameworks†

**DOI:** 10.1039/c9sc05757a

**Published:** 2019-12-16

**Authors:** Tomoya Itakura, Hiroshi Matsui, Tomofumi Tada, Susumu Kitagawa, Aude Demessence, Satoshi Horike

**Affiliations:** DENSO Corporation 1-1, Showa-cho Kariya Aichi 448-8661 Japan; Department of Physics, Graduate School of Science, Tohoku University 6-3, Aramaki Aza-Aoba, Aoba-ku Sendai 980-8578 Japan; Materials Research Center for Element Strategy, Tokyo Institute of Technology Nagatsuta-cho, Midori-ku Yokohama Kanagawa 226-8501 Japan; Institute for Integrated Cell-Material Sciences, Institute for Advanced Study, Kyoto University Yoshida-Honmachi, Sakyo-ku Kyoto 606-8501 Japan horike@icems.kyoto-u.ac.jp; Université Claude Bernard Lyon 1, Institut de Recherches sur la Catalyse et l'Environnement de Lyon (IRCELYON), UMR 5256 CNRS Villeurbanne France; AIST-Kyoto University Chemical Energy Materials Open Innovation Laboratory (ChEM-OIL), National Institute of Advanced Industrial Science and Technology (AIST) Yoshida-Honmachi, Sakyo-ku Kyoto 606-8501 Japan; Department of Synthetic Chemistry and Biological Chemistry, Graduate School of Engineering, Kyoto University Katsura, Nishikyo-ku Kyoto 615-8510 Japan; Department of Materials Science and Engineering, School of Molecular Science and Engineering, Vidyasirimedhi Institute of Science and Technology Rayong 21210 Thailand

## Abstract

We studied the relationship between proton conductivity and the terahertz-regime vibrations of two-dimensional MOFs. The results of spectroscopy studies clarified the essential role played by the collective motions in the terahertz region in 2D layers for efficient H^+^ conduction. *Ab initio* calculations suggested the collective motion to be predominantly determined by the valence electronic structure, depending on the identity of the metal ion.

## Abstract 2

Terahertz-regime vibrations of 2D MOFs dominate the intrinsic proton conductivity, and the motions depend on the valence electronic structures.

## Graphical abstract

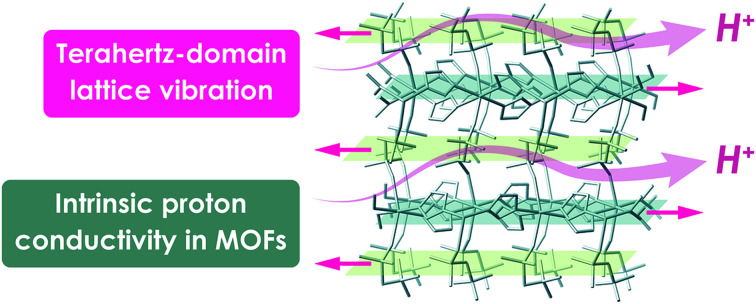

Solid state ion conductors are in wide demand for batteries, fuel cells, electrochemical sensors and electrochemical catalysis. The design and elucidation of ionically conductive paths in solid structures have, however, been significant challenges in relation to these applications.^1^ Ion conduction generally proceeds as a result of ions hopping to neighboring coordination sites, followed by local structural relaxation. These processes are often predominantly determined by lattice vibrations below 10 THz (333 cm^−1^).^2,3^ Indeed, ion conductivity in some representative inorganic materials correlates with the terahertz vibration. For α-AgI, for example, Ag^+^ behaves as a liquid and conducts as a consequence of sub-lattice melting with corresponding sub-lattice vibration modes of about 5–30 cm^−1^.^4^ For the typical proton (H^+^) conductors CsHSO_4_ and BaCeO_3_, the collective vibration modes of protonic species (*i.e.*, HSO_4_^−^ and lattice OH^−^, respectively) have been observed at 58–170 cm^−1^ and 320–378 cm^−1^.^2^

Studies of ion conductivity for molecular-based crystals have been recently showing rapid progress.^5^ Of such crystals, metal–organic frameworks (MOFs) are a promising class of materials for obtaining different ion conductivities.^6^ Researchers have shown the ability to control the ion conductivity by making use of guests in the channels, redox activity in metal centers, and ligand dynamics or acidity/basicity in the frameworks. Although there are many reports on the synthesis of ion-conductive MOFs, there are few about observations of any relationship between ion conductivity and lattice vibration. Several reports have described the unique lattice dynamics and functions of MOFs in the terahertz region,^7^ but it is also important to elucidate using spectroscopy the relationship between lattice dynamics and ion conductivity in MOFs. For this purpose, we employed three isostructural MOFs having two-dimensional (2D) H^+^-conductive pathways, and characterized their lattice vibrations in the terahertz region to describe their mechanism of H^+^ conductivity. We also carried out *ab initio* calculations to analyze vibrational mode characteristics and to study the contribution of metal ions to the lattice vibration.

We prepared three 2D MOFs having different metal ions as shown in Fig. 1. Each of the compounds may be described using the formula [M(H_2_PO_4_)_2_(TrH)_2_], where M = Zn^2+^, Co^2+^, or Mn^2+^, and TrH = 1,2,4-triazole, and we denote the compounds as ZnTr, CoTr, MnTr, respectively.^8^ The crystal structure of ZnTr is shown in Fig. 1A and B, and the parameters of the crystal structures of CoTr and MnTr are summarized in Table S1.† All three compounds crystallized in the same crystal system and space group (orthorhombic-*Pbcn*). H_2_PO_4_^−^ was observed to be coordinated to the metal ion axially to form a hydrogen bond network in the *ab* plane, with the extended H-bonds with H_2_PO_4_^−^ motion providing for the long-range hopping of H^+^ through the layer. The O(H)–O hydrogen bond distances between PO_4_ groups were measured to vary by less than 0.046 and 0.073 Å along the *a* and *b* directions, respectively. The X-ray results indicated negligible differences between the crystal structures of the three compounds.

**Fig. 1:**
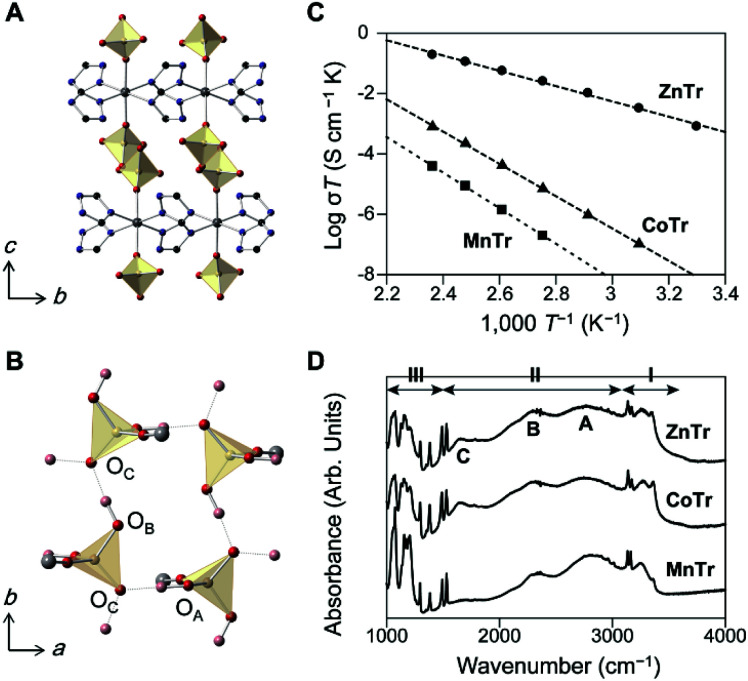
(A) The crystal structure of ZnTr along the *bc* plane. (B) The hydrogen-bond network formed by PO_4_ tetrahedra in ZnTr in the *ab* plane. Zn^2+^ and TrH are omitted for clarity. Zn: gray, P: yellow, O: red, C: black, N: blue, H: pink. (C) Plots of the H^+^ conductivity of ZnTr (circle), CoTr (triangle), and MnTr (square) under dry N_2_ gas from 30 to 150 °C. The dashed lines represent the fittings of the linear expression to the data. (D) Infrared spectra at 27 °C, with regions I and III attributed to intramolecular modes of TrH and PO_4_ tetrahedra, region II attributed to the O–H modes, band A to the O–H stretching mode, and bands B and C to the Fermi resonance with combinations involving O–H bending modes.

The ratio of the amount of H_2_PO_4_^−^ to that of metal ion was determined using ICP-AES for each of ZnTr, CoTr, and MnTr and found to be 1.58, 1.63, and 1.55. The lower value of the measured relative amount of H_2_PO_4_^−^ compared with the stoichiometric amount in the crystal structure was attributed to H_2_PO_4_^−^ site defects as described previously.^9^ These results also indicated the quantities of structural defects for the three compounds to be comparable. Therefore, the H^+^ conductivities for these structures were attributed to the mobility levels of the H_2_PO_4_^−^ groups in the layers.

Fig. 1C shows the plots of anhydrous H^+^ conductivity under an N_2_ atmosphere. The conductivity of ZnTr was found to be the highest: the values at 150 °C were measured to be 4.6 × 10^−4^ (ZnTr), 1.9 × 10^−6^ (CoTr) and 9.1 × 10^−8^ S cm^−1^ (MnTr). The total activation energy of ZnTr was determined to be 0.50 eV, about half of those of CoTr and MnTr (1.06 and 1.17 eV). The considerable differences between the H^+^ conductivities of these MOFs were found despite the near identities of their crystal structures and compositions, implying the predominant influence of other factors on the conductivity.

Infrared (IR) spectroscopy measurements at 27 °C were taken to investigate the characteristics of the hydrogen bonds (Fig. 1D). Regions I and III were attributed to intramolecular modes of TrH and PO_4_ tetrahedra, and region II to O–H modes. We assigned three broad bands, known as ABC bands and observed here from 1600 to 3100 cm^−1^ to the O–H modes, specifically to O–H stretching modes in Fermi resonance with combinations involving O–H bending mode, and are typically seen in strong hydrogen-bonding systems such as metal dihydrogenphosphates.^10^ Although two kinds of hydrogen bonds in MTr were in theory identified from the crystal structure, we could not distinguish between them due to small difference between their O–O distances. These O–H modes were indicated to be identical in their band shapes and frequencies, consistent with the results of their similar O–O distances from single crystal XRD (Table S1†). The IR spectroscopy results did not provide information about any distinct H^+^ conductivity.

To investigate the lattice vibration, we performed terahertz time-domain spectroscopy (THz-TDS) at 27 °C (Fig. 2A–C). We also computed the theoretical spectra using *ab initio* quantum mechanical calculations to identify the vibration modes (dotted lines and bars in Fig. 2A–C). The calculations were performed within the framework of density functional theory (DFT) using the VASP package.^11^ Vibrational properties were calculated using the linear response method of density functional perturbation theory (DFPT).^12^ For each normal mode, displacements of each of the ions and frequencies were calculated at the Brillouin zone center in a harmonic approximation. The theoretical spectra matched well with the experimental ones, although the frequencies were *ca.* 10 cm^−1^ higher.

**Fig. 2:**
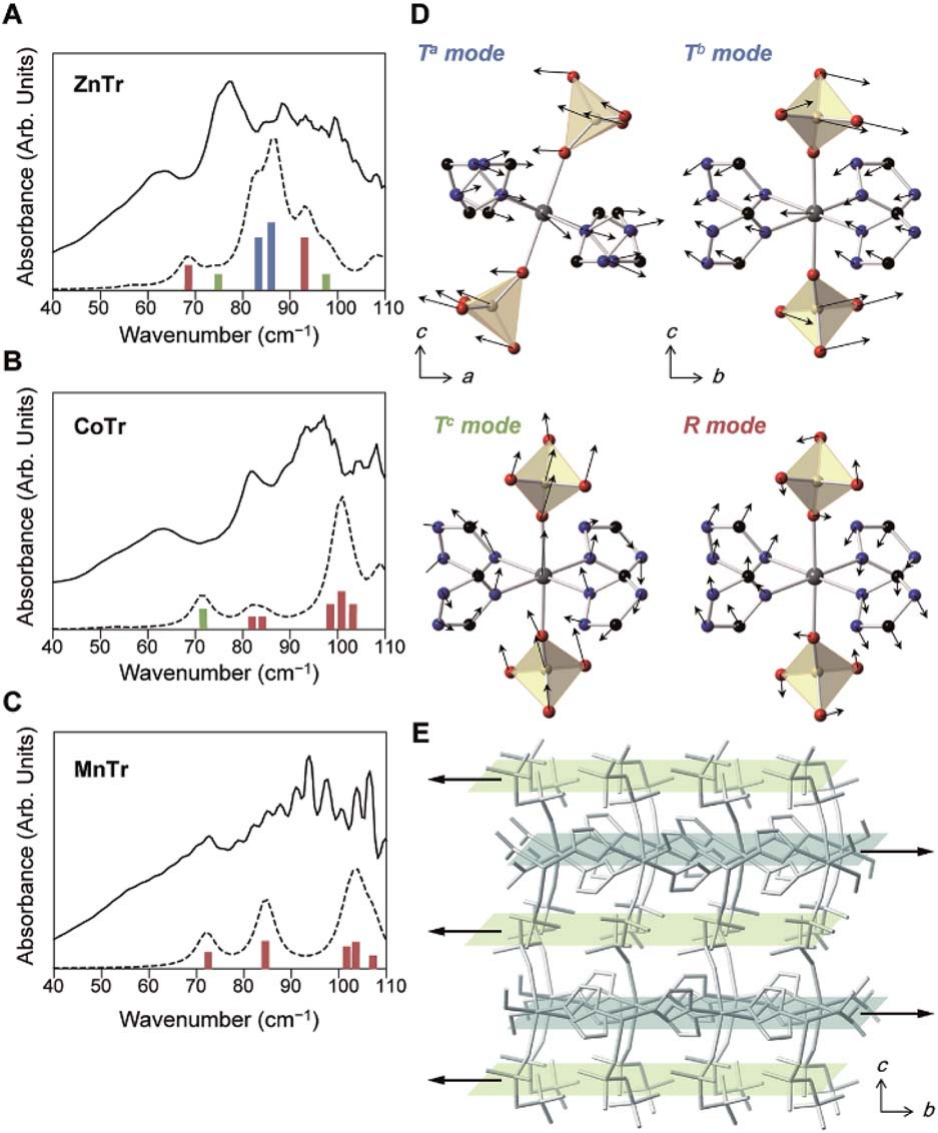
(A–C) THz-TDS spectra of (A) ZnTr, (B) CoTr, and (C) MnTr at 27 °C. The dotted lines are spectra obtained using DFT, applied to a 5 cm^−1^ FWHM Lorentzian-shaped line. The colors of the bars represent the types of motions of the PO_4_ tetrahedra: *R* mode (red), *T*^a^ and *T*^b^ mode (blue), and *T*^c^ mode (green). (D) A schematic illustration of each collective vibrational mode for the PO_4_ tetrahedra. The calculated eigenvectors of each normal mode for ZnTr are indicated by arrows. Shown are the PO_4_ translational mode along the *a* axis (upper left, *T*^a^ mode; 86.6 cm^−1^), PO_4_ translational mode along the *b* axis (upper right, *T*^b^ mode; 82.3 cm^−1^), PO_4_ translational mode along the *c* axis (lower left, *T*^c^ mode; 73.8 cm^−1^), and PO_4_ rotational mode (lower right, *R* mode; 68.5 cm^−1^). The color scheme is zinc = gray, phosphorous = yellow, oxygen = red, carbon = black, and nitrogen = blue. Hydrogens are omitted for clarity. (E) A schematic illustration of the *T*^b^ mode for the ZnTr periodic structure.

The optimized cell constants, which were used for DFPT, were found to be a few percent larger than the experimental ones (Table S2†), and this overestimate was attributed to the anharmonicity of the lattice vibrations of the MOFs or the limits of the exchange-correlation treatment in DFT.^13^ The increase of the baseline in the experimental spectra indicated the occurrence of considerable absorption at higher frequencies. In contrast to the IR results, the shapes of the THz-TDS spectra differed for the different samples tested, which suggested different lattice vibrations or intermolecular vibrations. We assigned the motions of each of the peaks by analyzing the eigenvector of each of the normal modes. With regards to the motions of the PO_4_ tetrahedron in H_2_PO_4_^−^, related to structural dynamics of the hydrogen bond network and H^+^ conduction, we found three types of such motions. The typical modes are illustrated in Fig. 2D. They are collective rotational (*R*; red bar), *a*- or *b*-directing translational (*T*^a^ or *T*^b^; blue bar) and *c*-directing translational (*T*^c^; green bar) modes, shown in Fig. 2A–C. The rotational mode represents each ion forming a PO_4_ tetrahedron moving in a rotating direction, and the translational mode represents each ion moving in the same direction parallel to the hydrogen-bond network for *T*^a,b^, but perpendicular for *T*^c^. In the ZnTr spectra, a large absorption band was observed at 70 to 80 cm^−1^ and consisted of two vibrational modes, which were assigned to *T*^a^ and *T*^b^. The other bands were assigned to *T*^c^ and *R*. In the cases of CoTr and MnTr, vibrational modes of PO_4_ were composed of *T*^c^ and *R*, and importantly, *T*^a^ and *T*^b^ modes were not observed in this frequency region. Thus, ZnTr, which exhibited the highest H^+^ conductivity, showed all PO_4_ modes in the terahertz region where we measured, with this feature increasing the degrees of freedom for PO_4_, resulting in the formation of an imperfect hydrogen-bond network and enhancing the mobility of H^+^. We also acquired THz-TDS spectra at various temperatures (27 to 107 °C, Fig. S2†) in order to investigate the dynamic disorder of the H_2_PO_4_^−^ in the lattice. The dependence of full width at half-maximum (FWMH) with temperature is shown in the figure. The bands corresponding to PO_4_ modes of ZnTr broadened and became more sensitive to temperature than did those of CoTr, which indicated that the dynamic disorder of PO_4_ increased in ZnTr upon heating. This behavior was reported for other inorganic H^+^ conductors such as solid acids, CsHSeO_4_ and CsH_2_PO_4_, which undergo a phase transition to that displaying superionic conductivity.^14^ The bands of collective XO_4_ modes (X = Se, P) including those of translational and rotational motions have been reported to broaden considerably as a result of the transition into the superionic phase. The lattice modes *T*^a^ and *T*^b^ in ZnTr, not observed in CoTr and MnTr, were indicated to be accompanied by a large displacement of Zn^2+^ in the *ab* plane, and also by a large distortion of the O–Zn^2+^–O bond angle—leading to oscillations of hydrogen-bonded network layers composed of H_2_PO_4_^−^ and Zn^2+^-TrH layers in opposite direction as shown in Fig. 2E.

To reveal the origin of the different lattice vibrations of the different MTr samples, we evaluated their metal–O bond characteristics using crystal-orbital Hamiltonian population (COHP) analysis.^15^ The COHP is a bond-weighted density of states between two adjacent atoms, and is calculated based on the corresponding Hamiltonian elements. It shows the number of states with which electrons participate in a covalent bond. Negative and positive COHP signs indicate, respectively, bonding and an anti-bonding states, and the absolute value of integrated COHP (ICOHP) indicates the covalent bond strength. Here, we determined the COHP absolute value of the single metal–O pair in each of the three samples to evaluate the covalency (Fig. 3A–C). In the Zn^2+^–O bond of ZnTr, the valence state was found to be significantly lower than the Fermi level. And the absolute value of ICOHP was calculated to be 3.19 × 10^−4^ eV, quite a bit lower than those of Co^2+^–O (2.46 eV) and Mn^2+^–O (9.43 × 10^−1^ eV) and hence indicating the highly ionic nature of the Zn^2+^–O interaction but covalent nature of the Co^2+^–O and Mn^2+^–O interactions. The valence states for CoTr and MnTr were determined to originate from the s, p and d orbitals of metal ions, with the states near the Fermi level in particular having strong d-character and being partially occupied.

**Fig. 3:**
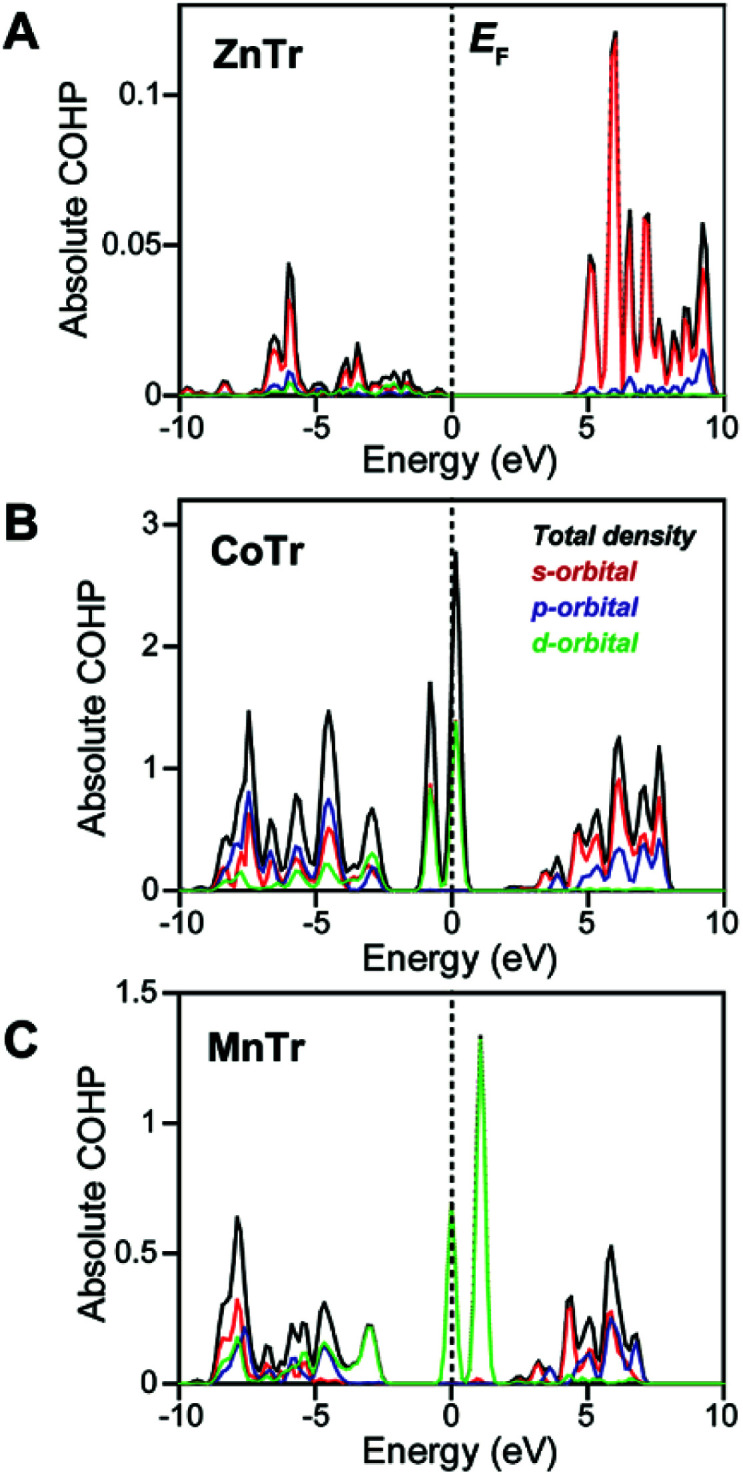
Bond characteristics from *ab initio* calculations. The absolute values of COHP of single metal–O pairs for (A) ZnTr, (B) CoTr, and (C) MnTr. The Fermi level (*E*_F_) was set at zero energy. Total density (black) is shown together with the contributions from the s (red), p (blue), and d orbitals (green) of the metal ion.

These states were attributed to molecular orbitals with e_g_ and t_2g_ symmetries formed from interactions between d orbitals of Co^2+^ and Mn^2+^ split in octahedral crystal fields and π-donating H_2_PO_4_^−^ ligand orbitals of the same symmetry. Thus, the covalent natures of the Co^2+^–O and Mn^2+^–O bonds were expected to suppress random mobility of the H_2_PO_4_^−^ ligands. On the other hand, the energy levels of the fully occupied d orbitals of Zn^2+^ were indicated to be localized at about −3.5 eV and to not interact with ligand orbitals of neighboring oxygen atoms in H_2_PO_4_^−^ (Fig. S3†). The valence state of Zn^2+^ consisting mainly of s-orbital character with a_1g_ symmetry indicated little orbital interaction between Zn^2+^ and H_2_PO_4_^−^ and hence the Zn–O bond to be ionic. Owing to the isotropic nature of the ionic bond, Zn^2+^ could easily change its coordination geometry, and hence allow the large distortions in O–Zn^2+^–O bond angle as seen in *T*^a^ and *T*^b^ modes. In other words, the limited lattice vibrations in CoTr and MnTr in the terahertz energy region were expected to prevent the free hopping of H^+^ attributed to the structural relaxation, resulting in the high activation energies (over 1.0 eV). The combination of THz-TDS and COHP analyses revealed the unique and preferential H^+^ conductivity of ZnTr compared with those of CoTr and MnTr.

## Conclusions

We applied terahertz time-domain spectroscopy (THz-TDS) to elucidate the origin of H^+^ conductivity in 2D MOFs in relation to their intrinsic lattice dynamics. H^+^ conductivity was concluded to be exclusively determined by the terahertz vibration modes in the 2D structures, and the vibration behaviors were concluded to depend on the connectivity of the coordination bonds of metal ions and ligands. COHP analysis suggested the ionic bonding character in the Zn^2+^-based framework to be key for achieving high H^+^ conduction, due to the ionic nature allowing the display of more isotropic vibration modes in the framework and hence facilitating efficient H^+^ hopping. This result, for the first time to the best of our knowledge, showed the lattice vibration of MOFs in the terahertz region to be strongly correlated with H^+^ conductivity. This result was also complementary with other characterizations including AC impedance spectroscopy in accounting for the origin of H^+^ conductivity in MOFs. Since we could modulate the unique vibration modes of MOFs, we expect the work described here to encourage the pursuit of further discoveries or structural optimizations of ion-conductive MOFs and other molecular framework materials.

## Conflicts of interest

There are no conflicts to declare.

## Supplementary Material

SC-011-C9SC05757A-s001
