# Supplementary material for: The role of lattice vibration in the terahertz region for proton conduction in 2D metal–organic frameworks
Source: Chem Sci. 2019 Dec 16;11(6):1538–41. doi: 10.1039/c9sc05757a (PMC8148082; doi:10.1039/c9sc05757a)
Supplement: SC-011-C9SC05757A-s001 [file SC-011-C9SC05757A-s001.pdf]

Electronic Supplementary Material (ESI) for Chemical Science.
This journal is © The Royal Society of Chemistry 2019

Supporting Information for

Role of Lattice Vibration in Terahertz-region for Proton Conduction in 2D Metal-organic Frameworks

Tomoya Itakura,[a] Hiroshi Matsui,*[b] Tomofumi Tada,[c] Susumu Kitagawa,[d] Aude Demessence,[e] and Satoshi Horike*[d,f,g,h]

[a] DENSO CORPORATION, 1-1, Showa-cho, Kariya, Aichi 448-8661, Japan

[b] Department of Physics, Graduate School of Science, Tohoku University, 6-3, Aramaki Aza-Aoba, Aoba-ku, Sendai 980-8578, Japan

[c] Materials Research Center for Element Strategy, Tokyo Institute of Technology, Nagatsuta-cho, Midori-ku, Yokohama, Kanagawa 226-8501, Japan

[d] Institute for Integrated Cell-Material Sciences, Institute for Advanced Study, Kyoto University, Yoshida-Honmachi, Sakyo-ku, Kyoto 606-8501, Japan

[e] Université Claude Bernard Lyon 1, Institut de Recherches sur la Catalyse et l'Environnement de Lyon (IRCELYON), UMR 5256 CNRS, Villeurbanne, France

[f] AIST-Kyoto University Chemical Energy Materials Open Innovation Laboratory (ChEM-OIL), National Institute of Advanced Industrial Science and Technology (AIST), Yoshida-Honmachi, Sakyo-ku, Kyoto 606-8501, Japan

[g] Department of Synthetic Chemistry and Biological Chemistry, Graduate School of Engineering, Kyoto University, Katsura, Nishikyo-ku, Kyoto 615-8510, Japan

[h] Department of Materials Science and Engineering, School of Molecular Science and Engineering, Vidyasirimedhi Institute of Science and Technology, Rayong 21210, Thailand

Materials

Zinc oxide was purchased from Sigma Aldrich Co., Ltd. 1,2,4-triazole, $Co(CH_3COO)_2{\cdot}4H_2O$, $Mn(CH_3COO)_2{\cdot}4H_2O$, phosphoric acid and ethanol were purchased from Wako Pure Chemical Industries Co., Ltd. All reagent was reagent grade and used without further purification.

Synthesis

Powder sample of $[M(H_2PO_4)_2(TrH)_2]$ (ZnTr, CoTr, MnTr) were synthesized as reported previously.[1] As a typical procedure for ZnTr, ZnO (1 mmol, 81 mg) and phosphoric acid (85% in H2O, 2 mmol, 136 μL) and 1,2,4-triazole (2 mmol, 138 mg) are put into a 10 mL Teflon jar with two Teflon balls with 10 mm diameter. The mixture was ground for 60 min in a Retch MM200 mixer mill at 25 Hz. The obtained white powder was washed with ethanol and dried at 80 °C for 15 h. The purity of each compound was checked by powder X-ray diffraction and Thermal gravimetric analysis (Figure S1). ICP-AES were used to determine the content of metal and phosphorous by SII NanoTechnology SPS-5100.

Conductivity measurement

AC impedance spectroscopy were carried out to determine the ion conductivity. Measurements were performed using impedance and gain-phase analyser (Solartron SI 1287/1255B) over frequency range 0.1 Hz to 1 MHz with an input voltage amplitude of 50 mV. Sample powders were pressed at 500 kg N for 2 minutes and sandwiched between carbon electrodes with 10 mm diameter, which were set into measurement cell that was filled with N_2 at 0.1 MPa. ZView software was used to fit impedance data sets by means of an equivalent circuit simulation to obtain the resistance values.

Spectroscopy

The infrared experiment (1,000-4,000 cm^{-1}) was performed by a Fourier-transform spectrometer (FT-IR 6100LT, JASCO) equipped with a Cassegrain microscope (IRT-5000, JASCO). The aperture size was fixed to be 100×100 μm^2, and the resolution was 4 cm^{-1}. Spectra were obtained at 27 °C for the samples mounted on an optical cryostat (ST-500, Cryogenic). The absorbance spectra in 40-110 cm^{-1} were obtained at 27-107 °C with a terahertz time-domain spectrometer (RT-20000, Tochigi Nikon). Time evolutions of transmitting electric field were measured for both the cases with and without the sample mounted on an aperture of optical cryostat. Each time evolution was converted into an intensity spectrum by Fourier transform. The difference between those intensity spectra provides the absorbance spectrum due to the sample.

Computational details

Total energies were computed from ab-initio quantum mechanical calculation within density functional theory (DFT) as implemented in VASP code (VASP 5.2).[2] We used the generalized gradient approximation by Perdew, Burke, and Ernzerhof for the exchange correlation energy[3]. The core electrons are handled in the projector augmented wave (PAW) method,[4] and valence electrons (H:$1s^1$, C:$2s^22p^2$, O:$2s^22p^4$, N:$2s^22p^5$, P:$3s^23p^5$, Zn:$3d^{10}4s^2$, Co:$3p^63d^84s^1$, Mn:$3p^63d^64s^1$) are represented with

wave functions based on plane waves. Energy cutoff for the plane-waves was set at 520 eV. The Monkhorst-Pack scheme with 4×4×2 *k*-points and total energy convergence of 0.8×10^{-6} eV per atoms were used. We performed optimization of the cell parameters and atomic positions. Spin-polarized calculations were executed for CoTr and MnTr in the structural relaxation, whereas spin-nonpolarized calculations were adopted for ZnTr. All atomic forces were reduced lower than 5 meV $Å^{-1}$. Table S4 lists the optimized cell parameters and structures of each model, and the optimized cell parameters of each model were in agreement to within 2.5% in the experimental data (Table S1). Vibrational calculations were carried out for the optimized structures within the density functional perturbation theory (DFPT) as implemented in the VASP code. The eigenvector and eigenvalues of normal modes were calculated in the harmonic approximation at the Brillouin zone center. All DFPT calculations were performed in spin-nonpolarized DFT approach, even though CoTr and MnTr are open-shell systems. We checked the validity of the vibrational spectra calculations by using the $Co(H_2PO_4)_2(TrH)_4$ cluster models which were relaxed by the spin-polarized and spin-nonpolarized calculation, respectively. As shown in Figure S4, there were no significant difference in the vibrational frequencies between them.

Table S1. X-ray crystallographic data and distance of hydrogen bonds (O(H)–O) for ZnTr, CoTr, and MnTr.

Parameters	**ZnTr**	**CoTr**	**MnTr**
Crystal system	Orthorhombic	Orthorhombic	Orthorhombic
Space group	*Pbcn*	*Pbcn*	*Pbcn*
a (Å)	8.4676(18)	8.4769(17)	8.7085(17)
b (Å)	9.549(2)	9.5314(19)	9.6808(19)
c (Å)	15.570(3)	15.479(3)	15.573(3)
α (°)	90.00	90.00	90.00
β (°)	90.00	90.00	90.00
γ (°)	90.00	90.00	90.00
V (Å^3)	1258.9(5)	1250.7(4)	1312.9(4)
Z	4	4	4
T (K)	223	293	173
GOF on F^2	1.210	1.108	1.091
No. of reflections measured	9318	8989	9788
No. of independent reflections	1442	1421	1497
R_1 ($I > 2\sigma$ (I))	0.0458	0.0265	0.0332
R_2 ($I > 2\sigma(I)$)	0.1454	0.0732	0.0869
O_A-O_B (Å)	2.552	2.549	2.593
O_B-O_C (Å)	2.566	2.550	2.623

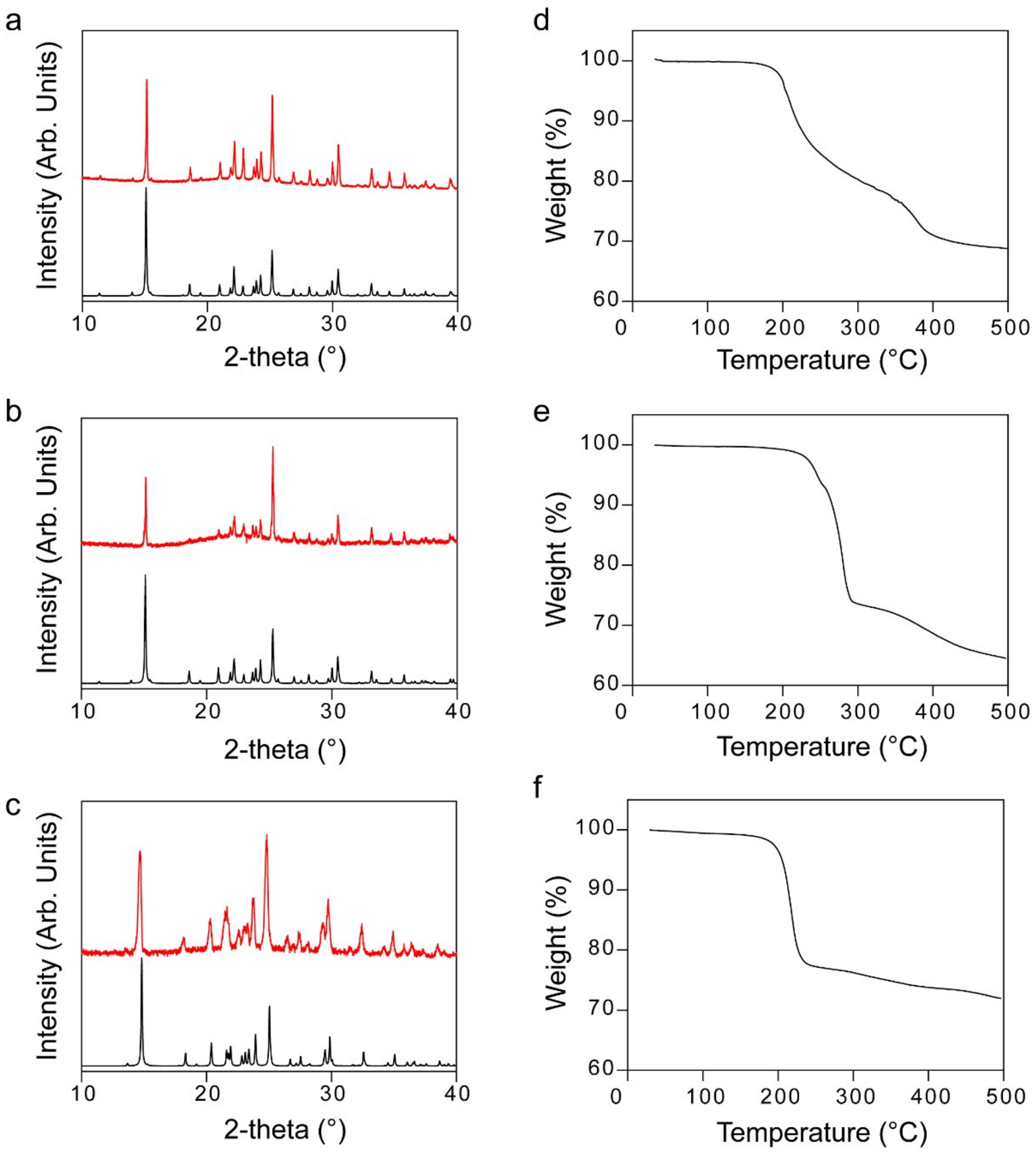

Figure S1. Powder X-ray diffraction (PXRD) patterns and thermal gravimetric (TG) profiles of ZnTr CoTr and MnTr. PXRD patterns of (a) ZnTr (b) CoTr (c) MnTr. Simulated patterns from the crystal structures and synthesized samples are shown in black and red, respectively. TG profiles of (d) ZnTr (e) CoTr (f) MnTr.

Table S2. Lattice parameters of the relaxed structures from DFT calculations.

	**ZnTr**			**CoTr**			**MnTr**		
	a	*b*	*c*	*a*	*b*	*c*	*a*	*b*	*c*
experimental (Å)	8.468	9.549	15.57	8.477	9.531	15.48	8.709	9.681	15.57
DFT (Å)	8.550	9.607	15.73	8.526	9.560	15.63	8.695	9.774	15.94
Error (%)	0.97	0.61	1.0	0.58	0.30	0.97	0.20	1.0	2.4

Table S3. Assignment of vibrational modes in terahertz time domain spectroscopy based on DFT calculations.

	**Wavenumber (cm^{-1})**	**Vibrational motions**
**ZnTr**	97.8 (ID_zn1)	O–Zn–O symmetric stretching; PO_4 tetrahedron translation along the *c*-axis (T^c-mode); TrH ring libration
	93.4 (ID_zn2)	O–Zn–O and P–O–Zn symmetric deformation; PO_4 tetrahedron rotation (*R* mode)
	86.6 (ID_zn3)	O–Zn–O symmetric deformation; PO_4 tetrahedron translation along the *a*-axis (T^a-mode)
	82.3 (ID_zn4)	O–Zn–O symmetric deformation; PO_4 tetrahedron translation along the *b*-axis (T^b-mode)
	73.8 (ID_zn5)	O–Zn–O asymmetric stretching; PO_4 tetrahedron translation along the *c*-axis (T^c-mode); TrH ring in-plane rotation
	68.5 (ID_zn6)	N–Zn–N asymmetric deformation; PO_4 tetrahedron rotation (*R* mode)
**CoTr**	101.2 (ID_co1)	O–Co–O asymmetric deformation and P–O–Co symmetric deformation; PO_4 tetrahedron rotation (*R* mode)
	100.9 (ID_co2)	O–Co–O symmetric deformation; PO_4 tetrahedron rotation (*R* mode); TrH ring libration
	99.9 (ID_co3)	O–Co–O and P–O–Co symmetric deformation; PO_4 tetrahedron rotation (*R* mode)
	84.9 (ID_co4)	N–Co–N asymmetric deformation; PO_4 tetrahedron rotation (*R* mode)
	81.6 (ID_co5)	O–Co–O asymmetric deformation; PO_4 tetrahedron rotation (*R* mode); TrH ring libration

	71.4 (ID_co6)	O–Co–O symmetric stretching; PO_4 tetrahedron translation along the *c*-axis (T^c-mode); TrH ring libration
**MnTr**	107.2 (ID_mn1)	O–Mn–O asymmetric deformation; PO_4 tetrahedron rotation (*R* mode); TrH ring libration
	103.8 (ID_mn2)	N–Mn–N asymmetric stretching; PO_4 tetrahedron rotation (*R* mode)
	101.8 (ID_mn3)	P–O–Mn symmetric deformation; PO_4 tetrahedron rotation (*R* mode); TrH ring libration
	84.6 (ID_mn4)	P–O–Mn symmetric deformation; PO_4 tetrahedron rotation (*R* mode); TrH ring libration
	72.1 (ID_mn5)	N–Mn–N asymmetric stretching; PO_4 tetrahedron rotation (*R* mode)

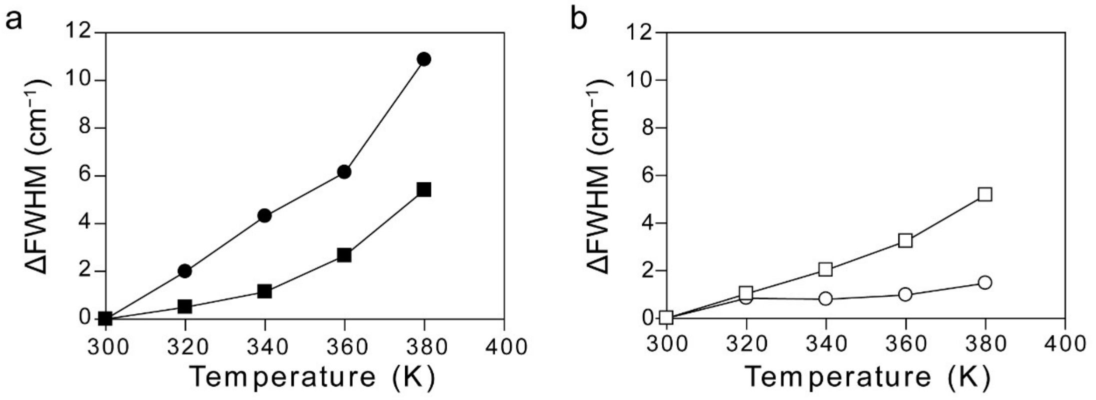

Figure S2. Temperature dependence of the vibrational line width in terahertz time domain spectra. (a) Temperature dependence of full width at half maximum (FWHM) of the 77 cm^{-1} (filled circle) including the T^a and T^b modes and 63 cm^{-1} (filled square) corresponding to the *R* mode in ZnTr. (b) Temperature dependence of FWHM of the 96 cm^{-1} (open square) corresponding to the *R* mode and 63 cm^{-1} (open circle) corresponding to the T^c mode in CoTr. The bands were fit with Lorentzian function to determine the FWHM. The vertical axis indicates the difference of FWHM with respect to the value at 300 K.

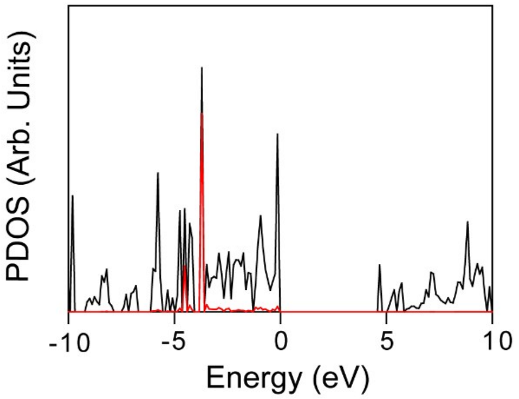

Figure S3. Partial Density of States (PDOS) of Zn^{2+} in ZnTr. Total and *d*-orbital density are shown with black and red line, respectively. The Fermi level is set at zero energy.

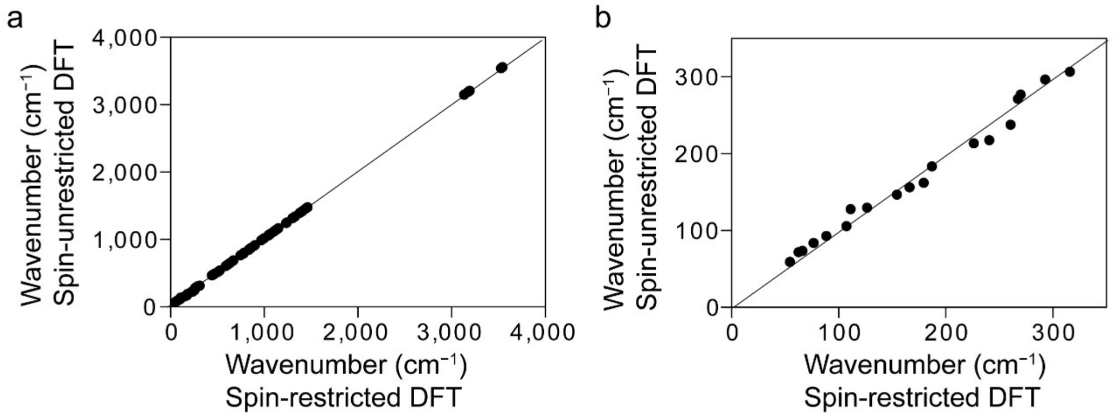

Figure S4. Comparison of vibrational wavenumbers between spin-restricted and spin-unrestricted DFT calculation. Calculations were performed for the cluster model of $Co(H_2PO_4)_2(TrH)_4$. The structure was cut from the periodic crystal structure of CoTr and then relaxed with the corresponding calculations. (a) All frequency region and (b) partially enlarged terahertz region (< 10 THz).

Table S4. Optimized cell parameters and structures of ZnTr, CoTr, and MnTr.

Optimized cell parameters of ZnTr:

8.5501443650461546	0.0000000000000000	0.0000000000000000
0.0000000000000000	9.6074069435741212	0.0000000000000000
0.0000000000000000	0.0000000000000000	15.7296881868551939

Optimized structure of ZnTr (fractional coordinate):

Zn	0.5000000000000000	0.2116309981891931	0.2500000920000005
Zn	0.0000000000000000	0.2883691478108048	0.7500002769999981
Zn	0.5000000000000000	0.7883692948108063	0.7500002769999981
Zn	0.0000000000000000	0.7116311451891946	0.2500000920000005
H	0.0245504644423633	0.9614054599395416	0.1053291911390772
H	0.3428636341477329	0.8910387734414442	0.3048232242184667
H	0.2179888046831380	0.1627588697549456	0.1132090079675905
H	0.7356481291385251	0.9648304517406885	0.0527637430039647
H	0.5053325749421589	0.2045791748710712	0.9577286576599917
H	0.4754495355576367	0.5385949810604629	0.6053293751390783
H	0.5245504644423633	0.5385949810604629	0.8946711778609213
H	0.9754495355576367	0.9614054599395416	0.3946709938609203
H	0.9754495355576367	0.0385948340604614	0.8946711778609213
H	0.5245504644423633	0.4614053129395401	0.3946709938609203
H	0.4754495355576367	0.4614053129395401	0.1053291911390772
H	0.0245504644423633	0.0385948340604614	0.6053293751390783
H	0.1571363658522671	0.6089616675585532	0.8048234082184678
H	0.8428636341477329	0.6089616675585532	0.6951771447815318
H	0.6571363658522671	0.8910387734414442	0.1951769607815308
H	0.6571363658522671	0.1089615205585517	0.6951771447815318
H	0.8428636341477329	0.3910386264414498	0.1951769607815308
H	0.1571363658522671	0.3910386264414498	0.3048232242184667
H	0.3428636341477329	0.1089615205585517	0.8048234082184678
H	0.2820111953168620	0.3372412772450559	0.6132091929675951
H	0.7179888046831380	0.3372412772450559	0.8867913610324081
H	0.7820111953168620	0.1627588697549456	0.3867911760324034
H	0.7820111953168620	0.8372414242450574	0.8867913610324081
H	0.7179888046831380	0.6627590167549471	0.3867911760324034
H	0.2820111953168620	0.6627590167549471	0.1132090079675905
H	0.2179888046831380	0.8372414242450574	0.6132091929675951
H	0.7643518708614749	0.5351699892593089	0.5527639270039657
H	0.2356481291385251	0.5351699892593089	0.9472366259960339
H	0.2643518708614749	0.9648304517406885	0.4472364419960329
H	0.2643518708614749	0.0351698422593145	0.9472366259960339
H	0.2356481291385251	0.4648303047406870	0.4472364419960329
H	0.7643518708614749	0.4648303047406870	0.0527637430039647
H	0.7356481291385251	0.0351698422593145	0.5527639270039657
H	0.9946674250578411	0.2954209721289374	0.4577284726599942
H	0.0053325749421589	0.2954209721289374	0.0422717113400068
H	0.4946674250578411	0.2045791748710712	0.5422718963400044
H	0.4946674250578411	0.7954211191289318	0.0422717113400068
H	0.0053325749421589	0.7045793218710728	0.5422718963400044
H	0.9946674250578411	0.7045793218710728	0.9577286576599917
H	0.5053325749421589	0.7954211191289318	0.4577284726599942

C	0.1208619371857438	0.9691190458858756	0.1499048367375408
C	0.2844041074598564	0.9359689473556827	0.2499978941029539
C	0.3791380628142562	0.5308813951141289	0.6499050217375384
C	0.6208619371857438	0.5308813951141289	0.8500955322624648
C	0.8791380628142562	0.9691190458858756	0.3500953472624602
C	0.8791380628142562	0.0308812481141274	0.8500955322624648
C	0.6208619371857438	0.4691188988858741	0.3500953472624602
C	0.3791380628142562	0.4691188988858741	0.1499048367375408
C	0.1208619371857438	0.0308812481141274	0.6499050217375384
C	0.2155958925401436	0.5640314936443218	0.7499980791029515
C	0.7844041074598564	0.5640314936443218	0.7500024748970446
C	0.7155958925401436	0.9359689473556827	0.2500022898970471
C	0.7155958925401436	0.0640313466443203	0.7500024748970446
C	0.7844041074598564	0.4359688003556812	0.2500022898970471
C	0.2155958925401436	0.4359688003556812	0.2499978941029539
C	0.2844041074598564	0.0640313466443203	0.7499980791029515
N	0.1575529714262132	0.8783965276756476	0.2114572668550920
N	0.3264525651569343	0.0564198089814738	0.2152169588643886
N	0.2209039057138540	0.0754424957321902	0.1519360757130883
N	0.3424470285737868	0.6216039133243569	0.7114574508550930
N	0.6575529714262132	0.6216039133243569	0.7885431021449065
N	0.8424470285737868	0.8783965276756476	0.2885429181449055
N	0.8424470285737868	0.1216037663243554	0.7885431021449065
N	0.6575529714262132	0.3783963806756461	0.2885429181449055
N	0.3424470285737868	0.3783963806756461	0.2114572668550920
N	0.1575529714262132	0.1216037663243554	0.7114574508550930
N	0.1735474348430657	0.4435803380185277	0.7152171428643896
N	0.8264525651569343	0.4435803380185277	0.7847834101356170
N	0.6735474348430657	0.0564198089814738	0.2847832261356160
N	0.6735474348430657	0.9435804850185221	0.7847834101356170
N	0.8264525651569343	0.5564199559814753	0.2847832261356160
N	0.1735474348430657	0.5564199559814753	0.2152169588643886
N	0.3264525651569343	0.9435804850185221	0.7152171428643896
N	0.2790960942861460	0.4245576512678113	0.6519362597130893
N	0.7209039057138540	0.4245576512678113	0.8480642932869102
N	0.7790960942861460	0.0754424957321902	0.3480641092869092
N	0.7790960942861460	0.9245577982678128	0.8480642932869102
N	0.7209039057138540	0.5754426427321917	0.3480641092869092
N	0.2790960942861460	0.5754426427321917	0.1519360757130883
N	0.2209039057138540	0.9245577982678128	0.6519362597130893
O	0.5811083500800294	0.2049595224158551	0.1200665952425624
O	0.9188916499199706	0.2950406245841464	0.6200667792425705
O	0.0811083500800294	0.2950406245841464	0.8799337737574291
O	0.4188916499199635	0.2049595224158551	0.3799335897574423
O	0.4188916499199635	0.7950407715841479	0.8799337737574291
O	0.0811083500800294	0.7049596684158530	0.3799335897574423
O	0.9188916499199706	0.7049596684158530	0.1200665952425624
O	0.5811083500800294	0.7950407715841479	0.6200667792425705
O	0.7986013659378628	0.0554941608100350	0.0517177845061596
O	0.8324207564928372	0.3117456795309437	0.0569057149739507
O	0.6263647859810249	0.2132668386282859	0.9603651459848308
O	0.7013986340621372	0.4445059861899665	0.5517179685061535
O	0.2986013659378628	0.4445059861899665	0.9482825844938461
O	0.2013986340621372	0.0554941608100350	0.4482824004938450

O	0.2013986340621372	0.9445061331899680	0.9482825844938461
O	0.2986013659378628	0.5554943078100365	0.4482824004938450
O	0.7013986340621372	0.5554943078100365	0.0517177845061596
O	0.7986013659378628	0.9445061331899680	0.5517179685061535
O	0.6675792435071628	0.1882544674690578	0.5569058989739517
O	0.3324207564928372	0.1882544674690578	0.9430946540260479
O	0.1675792435071557	0.3117456795309437	0.4430944700260468
O	0.1675792435071557	0.6882546134690557	0.9430946540260479
O	0.3324207564928372	0.8117458265309452	0.4430944700260468
O	0.6675792435071628	0.8117458265309452	0.0569057149739507
O	0.8324207564928372	0.6882546134690557	0.5569058989739517
O	0.8736352140189751	0.2867333083717156	0.4603649619848298
O	0.1263647859810249	0.2867333083717156	0.0396352230151749
O	0.3736352140189751	0.2132668386282859	0.5396354070151759
O	0.3736352140189751	0.7867334553717171	0.0396352230151749
O	0.1263647859810249	0.7132669856282874	0.5396354070151759
O	0.8736352140189751	0.7132669856282874	0.9603651459848308
O	0.6263647859810249	0.7867334553717171	0.4603649619848298
P	0.7057136923160812	0.1982341272521069	0.0520391363856803
P	0.7942863076839188	0.3017660197478875	0.5520393203856813
P	0.2057136923160812	0.3017660197478875	0.9479612326143183
P	0.2942863076839188	0.1982341272521069	0.4479610486143244
P	0.2942863076839188	0.8017661667478890	0.9479612326143183
P	0.2057136923160812	0.6982342742521084	0.4479610486143244
P	0.7942863076839188	0.6982342742521084	0.0520391363856803
P	0.7057136923160812	0.8017661667478890	0.5520393203856813

Optimized cell parameters of CoTr:

8.5263526267859078	0.0000000000000000	0.0000000000000000
0.0000000000000000	9.5601946062696506	0.0000000000000000
0.0000000000000000	0.0000000000000000	15.6321854979970514

Optimized structure of CoTr (fractional coordinate):

Co	0.0000000000000000	0.7898408502001638	0.2500000000000000
Co	0.0000000000000000	0.2101591497998365	0.7500000000000000
Co	0.5000000000000000	0.7101591497998362	0.7500000000000000
Co	0.5000000000000000	0.2898408502001636	0.2500000000000000
H	0.2369359963302091	0.0355478794255008	0.4454104014300959
H	0.7630640186697886	0.9644521205744994	0.5545896285699070
H	0.2630640186697883	0.4644521205744991	0.9454103714300930
H	0.7369359813302114	0.5355478794255006	0.0545895985699043
H	0.7630640186697886	0.0355478794255008	0.0545895985699043
H	0.2369359963302091	0.9644521205744994	0.9454103714300930
H	0.7369359813302114	0.4644521205744991	0.5545896285699070
H	0.2630640186697883	0.5355478794255006	0.4454104014300959
H	0.0009342722628701	0.7981341146399388	0.5411689712780552
H	-0.0009343002628724	0.2018658853600612	0.4588310287219443
H	0.4990657287371264	0.7018658853600612	0.0411689712780557
H	0.5009343002628724	0.2981341146399389	0.9588310287219448
H	-0.0009343002628724	0.7981341146399388	0.9588310287219448
H	0.0009342722628701	0.2018658853600612	0.0411689712780557

H	0.5009343002628724	0.7018658853600612	0.4588310287219443
H	0.4990657287371264	0.2981341146399389	0.5411689712780552
H	0.2827512285469692	0.8347348897584315	0.1145672623002642
H	0.7172487714530306	0.1652651102415684	0.8854327446997329
H	0.2172487714530305	0.6652651102415685	0.6145672553002671
H	0.7827512285469694	0.3347348897584316	0.3854327446997325
H	0.7172487714530306	0.8347348897584315	0.3854327446997325
H	0.2827512285469692	0.1652651102415684	0.6145672553002671
H	0.7827512285469694	0.6652651102415685	0.8854327446997329
H	0.2172487714530305	0.3347348897584316	0.1145672623002642
H	0.4769772229556721	0.0369568602620470	0.1065495090101756
H	0.5230227770443279	0.9630431397379527	0.8934504979898211
H	0.0230227770443279	0.4630431397379529	0.6065495020101789
H	0.9769772229556721	0.5369568602620473	0.3934504979898210
H	0.5230227770443279	0.0369568602620470	0.3934504979898210
H	0.4769772229556721	0.9630431397379527	0.6065495020101789
H	0.9769772229556721	0.4630431397379529	0.8934504979898211
H	0.0230227770443279	0.5369568602620473	0.1065495090101756
H	0.1525178462152563	0.1107699644305965	0.3041669284734259
H	0.8474821537847429	0.8892300355694035	0.6958330425265752
H	0.3474821537847437	0.3892300355694036	0.8041669574734248
H	0.6525178462152571	0.6107699644305965	0.1958330715265741
H	0.8474821537847429	0.1107699644305965	0.1958330715265741
H	0.1525178462152563	0.8892300355694035	0.8041669574734248
H	0.6525178462152571	0.3892300355694036	0.6958330425265752
H	0.3474821537847437	0.6107699644305965	0.3041669284734259
C	0.3795457948641264	0.0300350519888141	0.1508453499298336
C	0.6204541751358711	0.9699649480111859	0.8491546650701715
C	0.1204542051358735	0.4699649480111860	0.6508453349298285
C	0.8795458248641289	0.5300350519888141	0.3491546650701708
C	0.6204541751358711	0.0300350519888141	0.3491546650701708
C	0.3795457948641264	0.9699649480111859	0.6508453349298285
C	0.8795458248641289	0.4699649480111860	0.8491546650701715
C	0.1204542051358735	0.5300350519888141	0.1508453499298336
C	0.2128987434194562	0.0647366701956303	0.2499284497504652
C	0.7871012565805433	0.9352633298043692	0.7500715352495371
C	0.2871012565805440	0.4352633298043697	0.7499284647504629
C	0.7128987434194567	0.5647366701956308	0.2500715352495371
C	0.7871012565805433	0.0647366701956303	0.2500715352495371
C	0.2128987434194562	0.9352633298043692	0.7499284647504629
C	0.7128987434194567	0.4352633298043697	0.7500715352495371
C	0.2871012565805440	0.5647366701956308	0.2499284497504652
N	0.1719166923993055	0.9423936092440769	0.2156628281791514
N	0.8280833226006924	0.0576063907559231	0.7843372008208478
N	0.3280833226006924	0.5576063907559231	0.7156627991791522
N	0.6719166773993076	0.4423936092440766	0.2843371718208482
N	0.8280833226006924	0.9423936092440769	0.2843371718208482
N	0.1719166923993055	0.0576063907559231	0.7156627991791522
N	0.6719166773993076	0.5576063907559231	0.7843372008208478
N	0.3280833226006924	0.4423936092440766	0.2156628281791514
N	0.2795617569161738	0.9227486563489382	0.1529355703240475
N	0.7204382430838259	0.0772513436510617	0.8470644156759584
N	0.2204382430838262	0.5772513436510618	0.6529355843240416
N	0.7795617569161741	0.4227486563489382	0.3470644156759514

N	0.7204382430838259	0.9227486563489382	0.3470644156759514
N	0.2795617569161738	0.0772513436510617	0.6529355843240416
N	0.7795617569161741	0.5772513436510618	0.8470644156759584
N	0.2204382430838262	0.4227486563489382	0.1529355703240475
N	0.3412345726471520	0.1224299304880620	0.2119069988288645
N	0.6587653983528351	0.8775700695119378	0.7880929871711340
N	0.1587654273528478	0.3775700695119383	0.7119070128288660
N	0.8412346016471649	0.6224299304880622	0.2880929871711341
N	0.6587653983528351	0.1224299304880620	0.2880929871711341
N	0.3412345726471520	0.8775700695119378	0.7119070128288660
N	0.8412346016471649	0.3775700695119383	0.7880929871711340
N	0.1587654273528478	0.6224299304880622	0.2119069988288645
O	0.0778471087212425	0.7926772695016705	0.3775466220169355
O	0.9221528992787513	0.2073227304983296	0.6224533779830718
O	0.4221528992787510	0.7073227304983295	0.8775466220169282
O	0.5778471007212487	0.2926772695016705	0.1224533779830647
O	0.9221528992787513	0.7926772695016705	0.1224533779830647
O	0.0778471087212425	0.2073227304983296	0.8775466220169282
O	0.5778471007212487	0.7073227304983295	0.6224533779830718
O	0.4221528992787510	0.2926772695016705	0.3775466220169355
O	0.2979669580154862	0.9425700767922767	0.4449696170083077
O	0.7020330419845132	0.0574299232077236	0.5550303829916924
O	0.2020330419845138	0.5574299232077233	0.9449696170083076
O	0.7979669580154868	0.4425700767922763	0.0550303829916922
O	0.7020330419845132	0.9425700767922767	0.0550303829916922
O	0.2979669580154862	0.0574299232077236	0.9449696170083076
O	0.7979669580154868	0.5574299232077233	0.5550303829916924
O	0.2020330419845138	0.4425700767922763	0.4449696170083077
O	0.1221794024593362	0.7893440168504755	0.5382722162572267
O	0.8778206045406611	0.2106559831495237	0.4617277837427735
O	0.3778206045406609	0.7106559831495245	0.0382722162572266
O	0.6221793954593389	0.2893440168504765	0.9617277837427733
O	0.8778206045406611	0.7893440168504755	0.9617277837427733
O	0.1221794024593362	0.2106559831495237	0.0382722162572266
O	0.6221793954593389	0.7106559831495245	0.4617277837427735
O	0.3778206045406609	0.2893440168504765	0.5382722162572267
O	0.3282915292551079	0.6854594623572557	0.4431150323813021
O	0.6717084707448917	0.3145405376427447	0.5568849376187028
O	0.1717084707448921	0.8145405376427443	0.9431150623812972
O	0.8282915292551083	0.1854594623572553	0.0568849676186980
O	0.6717084707448917	0.6854594623572557	0.0568849676186980
O	0.3282915292551079	0.3145405376427447	0.9431150623812972
O	0.8282915292551083	0.8145405376427443	0.5568849376187028
O	0.1717084707448921	0.1854594623572553	0.4431150323813021
P	0.2028127885782438	0.8009566528953016	0.4463995396510220
P	0.7971872114217563	0.1990433471046985	0.5536004603489778
P	0.2971872114217561	0.6990433471046984	0.9463995396510222
P	0.7028127885782437	0.3009566528953012	0.0536004603489779
P	0.7971872114217563	0.8009566528953016	0.0536004603489779
P	0.2028127885782438	0.1990433471046985	0.9463995396510222
P	0.7028127885782437	0.6990433471046984	0.5536004603489778
P	0.2971872114217561	0.3009566528953012	0.4463995396510220

Optimized cell parameters of MnTr:

8.6951260024897810	0.0000000000000000	0.0000000000000000
0.0000000000000000	9.7743434750422278	0.0000000000000000
0.0000000000000000	0.0000000000000000	15.9404220744021874

Optimized structure of MnTr (fractional coordinate):

Mn	0.0000000000000000	0.7168157724959808	0.2500000000000000
Mn	0.0000000000000000	0.2831842275040191	0.7500000000000000
Mn	0.5000000000000000	0.7831842275040192	0.7500000000000000
Mn	0.5000000000000000	0.2168157724959809	0.2500000000000000
H	0.2311836510365923	0.4770901612312971	0.4449085065853278
H	0.7688163489634080	0.5229098387687026	0.5550914634146694
H	0.2688163489634074	0.0229098387687028	0.9449085365853306
H	0.7311836510365920	-0.0229098387687028	0.0550914934146721
H	0.7688163489634080	0.4770901612312971	0.0550914934146721
H	0.2311836510365923	0.5229098387687026	0.9449085365853306
H	0.7311836510365920	0.0229098387687028	0.5550914634146694
H	0.2688163489634074	-0.0229098387687028	0.4449085065853278
H	0.0063502852035851	0.7064745644021403	0.5375956813814430
H	-0.0063502682035872	0.2935254355978599	0.4624043186185570
H	0.4936497017964104	0.7935254355978597	0.0375956813814429
H	0.5063502682035876	0.2064745644021400	0.9624043186185570
H	-0.0063502682035872	0.7064745644021403	0.9624043186185570
H	0.0063502852035851	0.2935254355978599	0.0375956813814429
H	0.5063502682035876	0.7935254355978597	0.4624043186185570
H	0.4936497017964104	0.2064745644021400	0.5375956813814430
H	0.2825832757471683	0.6630226885476638	0.1097339403413077
H	0.7174166942528298	0.3369773114523355	0.8902660886586915
H	0.2174167242528321	0.8369773114523362	0.6097339113413085
H	0.7825833057471702	0.1630226885476643	0.3902660596586923
H	0.7174166942528298	0.6630226885476638	0.3902660596586923
H	0.2825832757471683	0.3369773114523355	0.6097339113413085
H	0.7825833057471702	0.8369773114523362	0.8902660886586915
H	0.2174167242528321	0.1630226885476643	0.1097339403413077
H	0.1593297598644711	0.3971763017840480	0.2991428128030981
H	0.8406702251355316	0.6028236982159517	0.7008571571968997
H	0.3406702251355310	0.1028236982159519	0.7991428428031003
H	0.6593297748644684	0.8971763017840483	0.2008571871969018
H	0.8406702251355316	0.3971763017840480	0.2008571871969018
H	0.1593297598644711	0.6028236982159517	0.7991428428031003
H	0.6593297748644684	0.1028236982159519	0.7008571571968997
H	0.3406702251355310	0.8971763017840483	0.2991428128030981
H	0.4716150318921535	0.4643106475387012	0.1019543405414198
H	0.5283849681078461	0.5356893224612966	0.8980456894585828
H	0.0283849681078461	0.0356893524612990	0.6019543105414172
H	0.9716150318921538	0.9643106775387034	0.3980456594585799
H	0.5283849681078462	0.4643106475387012	0.3980456594585799
H	0.4716150318921535	0.5356893224612966	0.6019543105414172
H	0.9716150318921539	0.0356893524612990	0.8980456894585828
H	0.0283849681078461	0.9643106775387034	0.1019543405414198
C	0.2168576843545531	0.4411245699855660	0.2449708400757598
C	0.7831423156454470	0.5588754600144291	0.7550291299242445
C	0.2831423156454471	0.0588754300144340	0.7449708700757555

C	0.7168576843545530	0.9411245399855709	0.2550291599242402
C	0.7831423156454470	0.4411245699855660	0.2550291599242402
C	0.2168576843545531	0.5588754600144291	0.7449708700757555
C	0.7168576843545530	0.0588754300144340	0.7550291299242445
C	0.2831423156454471	0.9411245399855709	0.2449708400757598
C	0.3769899180927360	0.4725347477061931	0.1459635770694305
C	0.6230100519072617	0.5274652822938032	0.8540364379305674
C	0.1230100819072637	0.0274652522938070	0.6459635620694326
C	0.8769899480927383	0.9725347177061969	0.3540364379305673
C	0.6230100519072617	0.4725347477061931	0.3540364379305673
C	0.3769899180927360	0.5274652822938031	0.6459635620694326
C	0.8769899480927384	0.0274652522938070	0.8540364379305674
C	0.1230100819072637	0.9725347177061969	0.1459635770694305
N	0.1753936879406461	0.5595609248603860	0.2107194857073014
N	0.8246063120593534	0.4404390751396142	0.7892805442927010
N	0.3246063120593538	0.9404390751396140	0.7107194557072990
N	0.6753936879406466	0.0595609248603860	0.2892805142926987
N	0.8246063120593534	0.5595609248603860	0.2892805142926987
N	0.1753936879406461	0.4404390751396141	0.7107194557072990
N	0.6753936879406466	0.9404390751396140	0.7892805442927010
N	0.3246063120593538	0.0595609248603860	0.2107194857073014
N	0.2790022633278024	0.5774592910986018	0.1479691642089002
N	0.7209977366721979	0.4225407089013988	0.8520308057910966
N	0.2209977366721977	0.9225407089013982	0.6479691942089034
N	0.7790022633278021	0.0774592910986014	0.3520308357911001
N	0.7209977366721979	0.5774592910986018	0.3520308357911001
N	0.2790022633278024	0.4225407089013988	0.6479691942089034
N	0.7790022633278021	0.9225407089013982	0.8520308057910966
N	0.2209977366721977	0.0774592910986014	0.1479691642089002
N	0.3408809032167128	0.3836912556876232	0.2068350891676902
N	0.6591191257832860	0.6163087143123755	0.7931649258323075
N	0.1591190967832873	0.1163087443123771	0.7068350741676925
N	0.8408808742167140	0.8836912856876245	0.2931649258323075
N	0.6591191257832860	0.3836912556876232	0.2931649258323075
N	0.3408809032167128	0.6163087143123755	0.7068350741676925
N	0.8408808742167140	0.1163087443123771	0.7931649258323075
N	0.1591190967832873	0.8836912856876245	0.2068350891676902
O	0.2939008497960477	0.5646505981258612	0.4448746173084188
O	0.7060991802039551	0.4353494018741383	0.5551253826915806
O	0.2060991502039522	0.9353494018741388	0.9448746173084194
O	0.7939008197960449	0.0646505981258616	0.0551253826915809
O	0.7060991802039551	0.5646505981258610	0.0551253826915809
O	0.2939008497960477	0.4353494018741383	0.9448746173084194
O	0.7939008197960449	0.9353494018741388	0.5551253826915806
O	0.2060991502039522	0.0646505981258616	0.4448746173084188
O	0.0879631266947906	0.7168379681564440	0.3755075381887870
O	0.9120368503052108	0.2831620318435558	0.6244924618112133
O	0.4120368803052064	0.7831620318435560	0.8755075381887867
O	0.5879631496947892	0.2168379681564442	0.1244924618112133
O	0.9120368503052108	0.7168379681564440	0.1244924618112133
O	0.0879631266947906	0.2831620318435558	0.8755075381887867
O	0.5879631496947892	0.7831620318435560	0.6244924618112133
O	0.4120368803052064	0.2168379681564442	0.3755075381887870
O	0.1236873357143723	0.7212030110862294	0.5340432101042654

O	0.8763126872856261	0.2787969889137704	0.4659567898957343
O	0.3763126572856306	0.7787969889137706	0.0340432101042655
O	0.6236873127143739	0.2212030110862298	0.9659567898957346
O	0.8763126872856261	0.7212030110862294	0.9659567898957346
O	0.1236873357143723	0.2787969889137704	0.0340432101042655
O	0.6236873127143739	0.7787969889137706	0.4659567898957343
O	0.3763126572856306	0.2212030110862298	0.5340432101042654
O	0.3335041684078924	0.8167306006922559	0.4432165155794950
O	0.6664958605921059	0.1832693993077440	0.5567835144205082
O	0.1664958315921077	0.6832693993077441	0.9432164855794918
O	0.8335041394078941	0.3167306006922558	0.0567834844205052
O	0.6664958605921059	0.8167306006922559	0.0567834844205052
O	0.3335041684078924	0.1832693993077440	0.9432164855794918
O	0.8335041394078941	0.6832693993077441	0.5567835144205082
O	0.1664958315921077	0.3167306006922558	0.4432165155794950
P	0.2065732162117980	0.7077995877490876	0.4445822600517807
P	0.7934268137882043	0.2922004122509119	0.5554177399482192
P	0.2934267837882018	0.7922004122509124	0.9445822600517808
P	0.7065731862117957	0.2077995877490882	0.0554177399482190
P	0.7934268137882043	0.7077995877490876	0.0554177399482190
P	0.2065732162117980	0.2922004122509119	0.9445822600517808
P	0.7065731862117957	0.7922004122509124	0.5554177399482192
P	0.2934267837882018	0.2077995877490882	0.4445822600517807

References

1. D. Umeyama, S. Horike, M. Inukai, T. Itakura and S. Kitagawa, *J. Am. Chem. Soc.*, 2012, **134**, 12780; Y. Ohara, A. Hinokimoto, W. Chen, T. Kitao, Y. Nishiyama, Y. L. Hong, S. Kitagawa and S. Horike, *Chem. Commun.*, 2018, **54**, 6859.
2. G. Kresse and J. Furthmuller, *Comp. Mater. Sci.*, 1996, **6**, 15.
3. J. P. Perdew, K. Burke and M. Ernzerhof, *Phys. Rev. Lett.*, 1996, **77**, 3865.
4. Blöchl, P. E. Projector augmented-wave method. *Phys. Rev. B* 1994, **50**, 17953.
